# Genetic Profiling of *Aggregatibacter actinomycetemcomitans* Serotype B Isolated from Periodontitis Patients Living in Sweden

**DOI:** 10.3390/pathogens8030153

**Published:** 2019-09-17

**Authors:** Anders Johansson, Rolf Claesson, Carola Höglund Åberg, Dorte Haubek, Mark Lindholm, Sarah Jasim, Jan Oscarsson

**Affiliations:** 1 Division of Molecular Periodontology, Department of Odontology, Umeå University, 907 00 Umeå, Sweden; 2 Division of Oral Microbiology, Department of Odontology, Umeå University, 907 00 Umeå, Sweden; 3 Section for Pediatric Dentistry, Department of Dentistry and Oral Health, Aarhus University, 8000 Aarhus, Denmark

**Keywords:** *Aggregatibacter actinomycetemcomitans*, *cagE*, *virB1*, *virB4*, genotype, virulence

## Abstract

The bacterium *Aggregatibacter actinomycetemcomitans* is associated with aggressive forms of periodontitis and with systemic diseases, such as endocarditis. By assessing a Ghanaian longitudinal adolescent cohort, we earlier recognized the *cagE* gene as a possible diagnostic marker for a subgroup of JP2 and non-JP2 genotype serotype b *A. actinomycetemcomitans* strains, associated with high leukotoxicity as determined in a semi-quantitative cell assay. This group of *A. actinomycetemcomitans* is associated with the progression of attachment loss. In the present work, we used conventional polymerase chain reaction (PCR) and quantitative PCR to perform the *cagE* genotyping of our collection of 116 selected serotype b *A. actinomycetemcomitans* strains, collected over a period of 15 years from periodontitis patients living in Sweden. The *A. actinomycetemcomitans* strains carrying *cagE* (referred to as *cagE*^+^; n = 49) were compared to the *cagE*-negative strains (n = 67), present at larger proportions in the subgingival plaque samples, and were also much more prevalent in the young (≤35 years) compared to in the old (>35 years) group of patients. Our present results underline the potential use of *cagE* genotyping in the risk assessment of the development of periodontal attachment loss in Swedish adolescents.

## 1. Introduction

*Aggregatibacter actinomycetemcomitans* is a Gram-negative opportunistic pathogen associated with rapidly progressing periodontitis and with extra-oral diseases, such as endocarditis [1,2,3]. Several longitudinal studies have demonstrated that adolescents colonized with *A. actinomycetemcomitans*, as compared to those that are not, have a significantly increased risk of the development of periodontal attachment loss (AL) [4,5,6,7]. *A. actinomycetemcomitans* produces an array of virulence factors that allow this bacterium to evade and suppress the host immune response, including two exotoxins, i.e., leukotoxin and cytolethal distending toxin (CDT) [8,9,10]. A large genetic diversity within the *A. actinomycetemcomitans* species has been found, and seven different serotypes (a–g) exist, representing genetically divergent lineages [11,12,13]. *A. actinomycetemcomitans* genotypes can have extensively different pathogenic potentials [5,14,15]. For example, carriers of the JP2 serotype b-specific genotype of *A. actinomycetemcomitans* are at higher risk of development of AL compared to carriers of a non-JP2 genotype of *A. actinomycetemcomitans*. Typical for JP2 genotype strains is the deletion of 530 base pairs (bp) in the promoter region of the *ltxCABD* gene operon, which encodes leukotoxin (LtxA), and an enhanced leukotoxicity [16,17]. LtxA is a virulence factor of *A. actinomycetemcomitans* with the capacity to cause imbalance in the host inflammatory response [9]. The *ltx* promoter deletion has been frequently used as genetic marker to identify *A. actinomycetemcomitans* carriers with an increased risk for periodontal disease onset and progression [17], and this genotype is easily detected using a DNA-based assay (PCR) [18]. In addition to the JP2 genotype, a subgroup of non-JP2 genotype serotype b strains exhibits a similar disease association and high leukotoxicity, as has been shown in a semi-quantitative cell assay [14]. Genetic characterization has revealed that this particular subgroup of non-JP2 genotype of *A. actinomycetemcomitans* strains of serotype b are genetically closely related to the JP2 genotype by sharing the same arbitrarily-primed (AP) PCR gel electrophoresis banding pattern, referred to as AP-PCR genotype 1 in the present work [14]. Another property shared between the JP2 genotype and highly leukotoxic non-JP2 genotype serotype b strains was recently recognized, i.e., the carriage of the *cagE* gene sequence [19]. The *cagE* gene in *A. actinomycetemcomitans* was initially characterized by Teng and Hu [20], presenting evidence that the encoded CagE protein could induce apoptosis on primary human epithelial cells. However, consistent with leukotoxicity being a major virulence property of *cagE*-positive *A. actinomycetemcomitans* serotype b strains, one JP2 genotype bacterial cell was enough to lyse the majority of macrophage cells in vitro [20], whereas, as in comparison, a ratio of 50,000 JP2 genotype bacterial cells per epithelial cell was used to detect the CagE-induced apoptotic effects in vitro [21]. This suggests that CagE may have limited overall contribution to the virulence at biologically-relevant bacterial levels. In the present study, we utilized the *cagE* gene sequence as a diagnostic risk marker for the PCR detection of highly leukotoxic JP2 and non-JP2 genotypes of *A. actinomycetemcomitans* serotype b [19]. 

Furthermore, a type IV secretion system (T4SS) is a large macromolecular complex in Gram-negative bacteria which mediates conjugation, DNA transport and the secretion of virulence factors. Experimental work on model organisms, such as *Agrobacterium tumefaciens* and *Helicobacter pylori*, has revealed an archetypal T4SS system composed of 12 proteins, referred to as VirB1–VirB11, and VirD4 [22,23]. *A. actinomycetemcomitans* T4SS gene clusters are found in approximately 50% of strains and can be encoded both on the chromosome and on plasmids [24,25,26]. Interestingly, CagE exhibits homology to two T4SS proteins. The CagE N-terminus is homologous to VirB1 (lytic transglycosylase; also known as MagB01), and the CagE C-terminus is homologous to VirB4 (ATP:ase; MagB03) [19]. As judged by in silico analysis of the serotype b genomes available in the National Center for Biotechnology (NCBI) database, the *cagE* gene locus is not present in any of the strains encoding VirB1 and VirB4 on the chromosome. Whether the *cagE* and *virB1*/*virB4* genes are consistently inversely carried in serotype b strains has not earlier been thoroughly assessed but would support the notion that CagE may represent the result of a recombination event in which parts of the *virB1* and *virB4* genes were fused together to encode a chimeric VirB1–VirB4 protein in *A. actinomycemcomitans* [19]. 

In our previous work, delineating the role of *cagE* as a potential diagnostic marker, we studied a collection of *A. actinomycetemcomitans* strains collected from a prospective cohort of Ghanaian adolescents [19]. To further evaluate the role of *cagE* and *virB1*/*virB4* as diagnostic tools, we assessed our collection of *A. actinomycetemcomitans* strains that were collected during 15 years from periodontitis patients living in Sweden [27]. Data from microbiological analyses of this collection revealed that the young individuals (≤35 years) had a higher prevalence of *A. actinomycetemcomitans* and larger proportions of it in the samples compared to the older patients (>35 years). Moreover, serotype b was highly prevalent in the samples collected from young patients [27]. The aim of the present work was to determine the prevalence of the *cagE* genotype among the serotype b strains from this aforementioned collection (n = 116) and also to evaluate the potential use of *cagE* as a diagnostic marker for the carriage of highly leukotoxic serotype b strains among periodontitis patients living in Sweden. 

## 2. Results 

### 2.1. Validation of PCR Assays to Detect VirB1 and VirB4 Sequences in A. actinomycetemcomitans Serotype B Reference Strains

**Figure 1:**
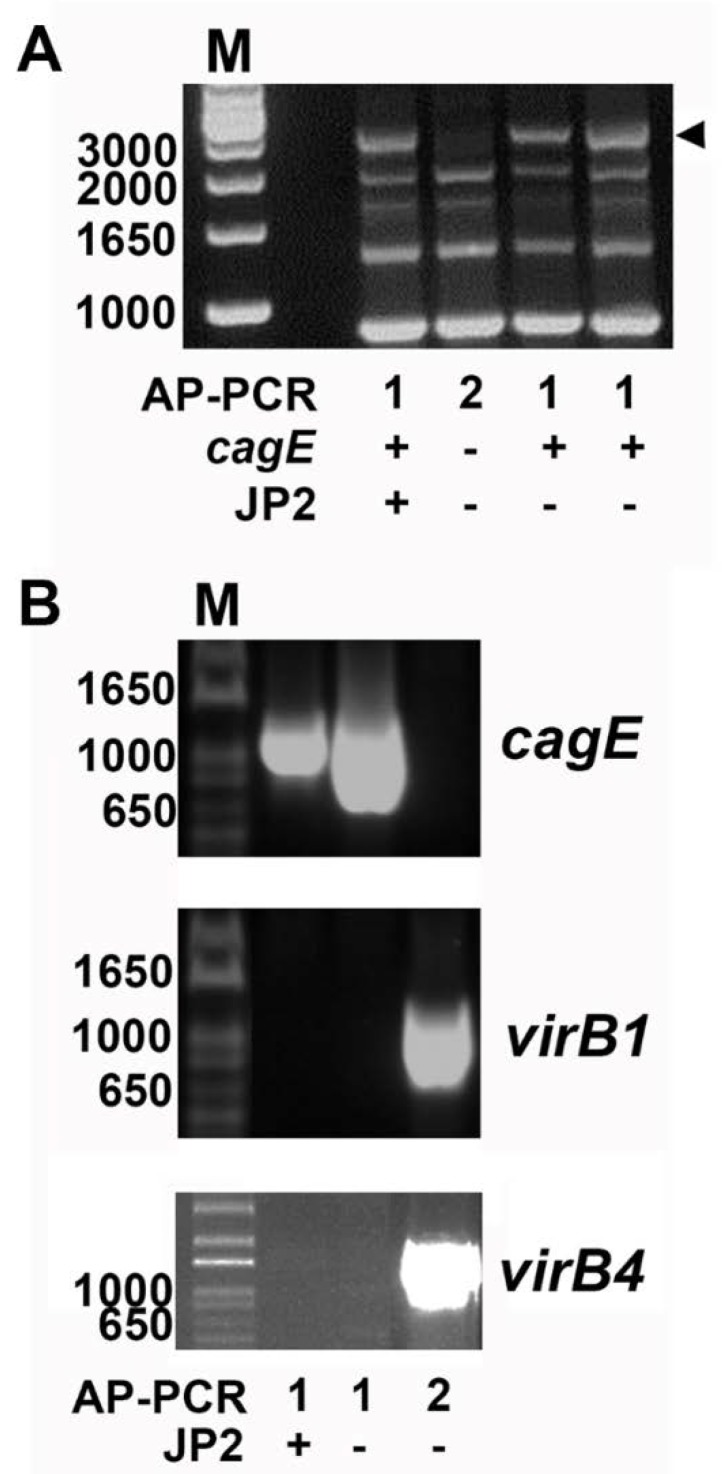
PCR genotyping of *Aggregatibacter* *actinomycetemcomitans* serotype b strains. (**A**) Distinct arbitrarily-primed (AP)-PCR banding patterns distinguish *cagE*-positive and *cagE*-negative serotype b strains of *A. actinomycetemcomitans*. The approximately 3000-bp DNA-band (arrowed) detected in AP-PCR type 1 is unique for this genotype and was earlier demonstrated to contain the *cagE* gene sequence [19]. Typically, this DNA band reflects the difference between AP-PCR types 1 and 2. The presence/absence of the *cagE* gene and *ltxA* JP2 promoter type in AP-PCR types 1 and 2 is indicated. (**B**) PCR detection of *cagE*, *virB1*, and *virB4*, respectively. An amplicon specific for *cagE* was revealed in both JP2 and non-JP2 AP-PCR genotype 1 strains. In AP-PCR genotype 2 strains, amplicons specific for *virB1* and *virB4* were detected, whereas *cagE* was not. Sizes (bp) of selected bands in the DNA molecular weight marker (M) are indicated. Figures illustrate representative experiments.

All A. *actinomycetemcomitans* serotype b strains assessed in the present study were grouped according to their AP-PCR genotype—1, 2 or “other” (i.e., AP-PCR types 3–11 as defined earlier [27]) (Figure 1A). 

To test the hypothesis that the presence of chromosomal *virB1* and *virB4* genes can serve as genetic markers that are suitable for the detection of *cagE*-negative serotype b strains, PCR was employed as described in the Materials and Methods section. To evaluate the PCR approach, we initially assessed 25 *A. actinomycetemcomitans* strains of serotype b which have previously been subject to whole genome sequencing (Table 1) (Figure 1B). As expected, this revealed presence of both *virB1* and *virB4* in the *cagE*-negative strains only (n = 7; 4 type 2 AP-PCR and 3 “other” AP-PCR type), whereas neither *virB1* nor *virB4* were detected by PCR in the *cagE*-positive strains (n = 18; all AP-PCR type 1). This finding prompted us to further investigate this apparent inverse relationship between the carriage of *cagE* and *virB1*/*virB4* in the assessment of our local collection of serotype b *A. actinomycetemcomitans* strains. As *virB1* and *virB4* were carried simultaneously in the strains studied, we continued our analyses, mainly screening for the presence of *virB4*.

### 2.2. Screening of CagE and VirB4 in Serotype B A. actinomycetemcomitans Strains Collected from Patients with Periodontitis Living in Sweden 

**Figure 2:**
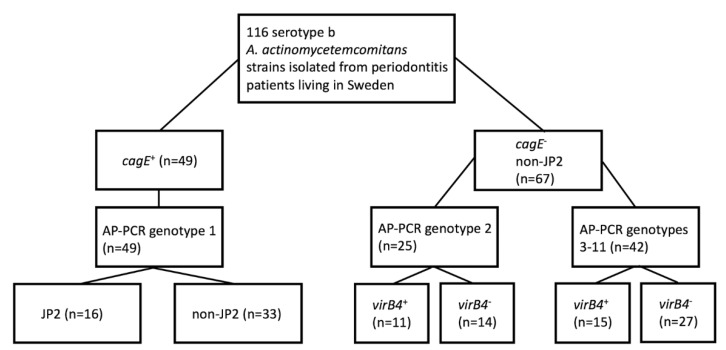
Genotype patterns of *A. actinomycetemcomitans* serotype b strains. Schematic overview of AP-PCR type, as well as the JP2- and *virB4*-genotype patterns of the *cagE*-positive and *cagE*-negative strains, respectively. The collection of 116 serotype b strains was earlier sampled from periodontitis patients living in Sweden [27].

We screened the 116 serotype b *A. actinomycetemcomitans* strains, collected from periodontitis patients living in Sweden, using qPCR to determine the prevalence of the *cagE* and *virB4* genes (Table 2) (Table S1) (Figure 2). 

According to our results, *cagE* was present in 49 (42.2%) strains, including all 16 JP2 genotype strains, and hence absent in 67 (57.8%) strains. Of the *cagE*-positive strains, all (100%) belonged to AP-PCR genotype 1. Interestingly, three *cagE*-positive strains (all non-JP2 genotypes) were found to carry the *virB4* gene. However, PCR analysis, using the primers *magB01*-F and *ssb*-R, supported that all three strains most likely carried *virB4* on a plasmid rather than on the chromosome (data not shown). Thus, we concluded that a property common among the *cagE*-positive strains is an apparent lack of a chromosomal *virB4* gene. Of the *cagE*-negative *A. actinomycetemcomitans* strains, 25 (37.3%) belonged to AP-PCR genotype 2, and 42 (62.7%) belonged to AP-PCR genotypes 3–11. The prevalence of *virB4* was somewhat higher among the AP-PCR genotype 2 *A. actinomycetemcomitans* strains (44%) compared to the strains belonging to AP-PCR genotypes 3–11 (35.7%), suggesting that *virB4* might be usable as a genetic marker for a subgroup of *cagE*-negative strains. Thus, taken together, as none of the 116 strains studied encoded both *cagE* and *virB4* on the chromosome, we concluded that there is an apparent inverse relationship in the carriage of these genes in the *A. actinomycetemcomitans* strains of serotype b. 

### 2.3. Higher Proportions of CagE-Positive A. actinomycetemcomitans Serotype B in Subgingival Plaque Samples 

**Figure 3:**
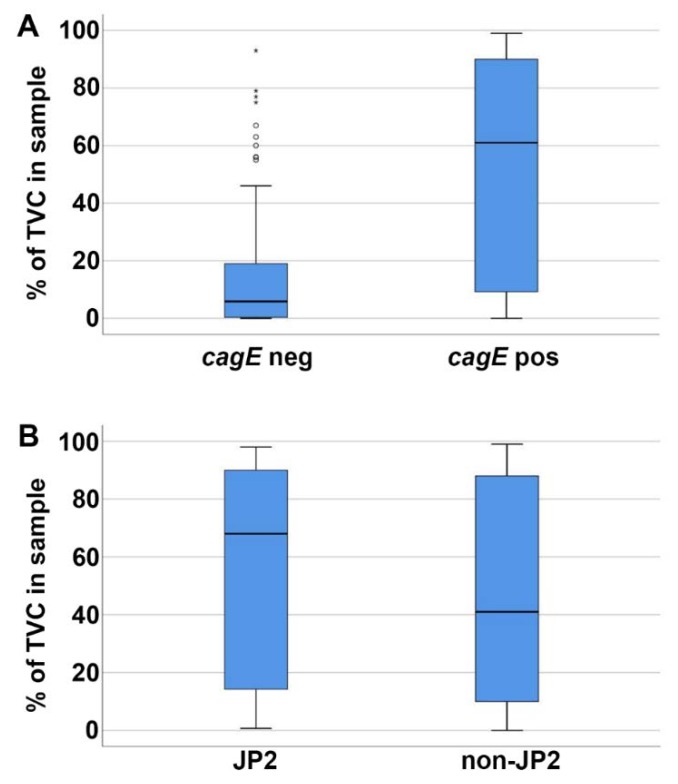
Higher proportions of *A. actinomycetemcomitans* in subgingival plaque samples containing a *cagE*-positive serotype b. The proportion of *A. actinomycetemcomitans* (total viable count—TVC; %) in the subgingival plaque samples was determined earlier for each of the serotype b strains (n = 116) [27]. (**A**) The *cagE*-positive strains (n = 49) were present in significantly higher (*p* < 0.001) proportions than the *cagE*-negative strains (n = 67). (**B**) The JP2 (n = 16) and non-JP2 (n = 33) strains were present at similar proportions. Median and quartiles from the samples are shown in each panel.

Furthermore, we assessed whether the *cagE* genotype may correlate with the proportion of *A. actinomycetemcomitans* found in the subgingival plaque samples. For this, the serotype b *A. actinomycetemcomitans* strains (n = 116) were divided into two groups, i.e., *cagE*-positive (n = 49) and *cagE*-negative (n = 67), and then they were matched with the determined total viable counts (%) of *A. actinomycetemcomitans* in the respective samples [27]. This clearly revealed that *cagE*-positive strains were carried in patients at significantly higher (*p* < 0.001) proportions than *cagE*-negative *A. actinomycetemcomitans* (Figure 3A). However, among the *cagE*-positive, the proportion of *A. actinomycetemcomitans* in samples with a JP2 genotype strain (n = 16) was not significantly different from that with a non-JP2 genotype strain (n = 33) (Figure 3B). 

### 2.4. Higher Prevalence of CagE-Positive A. actinomycetemcomitans Serotype B in Young Patients 

To further evaluate the virulence of the *cagE*-positive serotype b *A. actinomycetemcomitans* strains (n = 49) among periodontitis patients living in Sweden, we also assessed the age-associated prevalence of these strains. For this purpose, the patients (n = 116) were grouped into young (≤35 years; n = 62) and old (>35 years; n = 54) groups (Table 3). This revealed that among the young patients, *cagE*^+^ *A. actinomycetemcomitans* strains (n = 40; 64.5%) were much more common than among the older patients (n = 9; 16.7%), i.e., these strains had a significantly higher (*p* < 0.001, odds ratio (OR) = 9.1, 95% CI: 3.8–22.0) prevalence among the young patients. 

## 3. Discussion

In the present work, we used conventional PCR and qPCR to genotypically analyze our collection of 116 *A. actinomycetemcomitans* serotype b strains, collected during 15 years from periodontitis patients living in Sweden, and our present results underline the potential use of *cagE* genotyping in the risk assessment of the development of periodontal attachment loss in adolescents living in Sweden.

Each of the 116 serotype b strains were matched both with its load (% of total viable count) in the respective subgingival plaque sample and with its age-associated prevalence category [27]. As *cagE*-positive, in contrast to *cagE*-negative serotype b *A. actinomycetemcomitans* strains, were found at larger proportions in the plaque samples and exhibited a much higher prevalence in the young compared to in the old patients of this population, our present results are consistent with our findings assessing the longitudinal Ghanaian adolescent cohort [19]. Whereas the proportions of *A. actinomycetemcomitans* genotypes in the total viable counts of subgingival plaque samples had not earlier been assessed in patient cohorts, the ratio of *cagE*-positive among serotype b strains carried by young patients in the Swedish population (64.5%) was similar to that of the adolescents in the Ghanaian cohort, exhibiting an association between the progression of attachment loss and exposure to this particular *cagE*-positive genotype [14,19]. As the *cagE*-positive serotype b strains sampled in both Ghana and Sweden were found at larger proportions in the plaque samples and exhibited a much higher prevalence in the young group of patients [19,27], the results from our present study are consistent with the notion that *cagE*-positive strains (including both the JP2 and non-JP2 genotypes) represent a subgroup of highly virulent *A. actinomycetemcomitans* serotype b.

**Figure 4:**
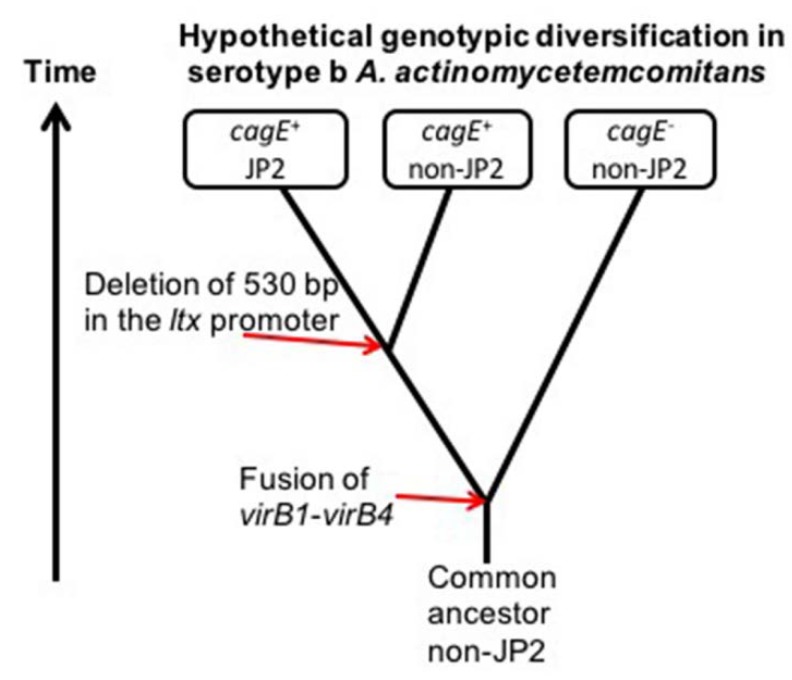
Hypothetical origin of the *cagE* and JP2 genotypes in serotype b *A. actinomycetemcomitans*. The genetic similarity between *cagE*-positive strains and the apparent absence of the JP2 genotype among *cagE*-negative strains suggests the possibility that the JP2-associated 530-bp deletion in the *ltx* promoter might have originated in a *cagE*^+^ strain. The JP2 genotype of *A. actinomycetemcomitans* initially appeared as a distinct genotype in the Mediterranean part of Africa approximately 2400 years ago [29]. As *cagE*^+^ strains consistently lack chromosomal copies of *virB1* and *virB4*, an earlier recombination event causing fusion of a *virB1*- and a *virB4*-like gene sequence resulting in the *cagE* determinant might have taken place in a common serotype b *A. actinomycetemcomitans* ancestral strain.

The genetic similarity of *cagE*^+^ serotype b strains is supported by the observation that they share the same AP-PCR genotype, as well as the fact that they have all a complete *cdtABC* gene operon [30]. We speculated earlier that they may belong to a clonal lineage that is closely related to the JP2 genotype ancestor [19]. As *cagE*-positive strains include both the JP2 and non-JP2 genotypes but no identified JP2-genotype strain has thus far been found to be *cagE*-negative, it is hypothesized that the JP2 genotype-associated deletion in the *ltxCABD* promoter once originated in a *cagE*-positive serotype b strain (Figure 4). It is tempting to speculate that high leukotoxicity may have been a characteristic of this ancestral *A. actinomycetemcomitans* strain, as that is a property common among *cagE*-positive strains, regardless of whether they are of the JP2 genotype or not. 

Consistent with our earlier in silico analysis of the genome-sequenced serotype b strains in the NCBI database [19], another property shared between the *cagE*-positive strains assessed in the present work was a lack of chromosomal genes encoding the T4SS-associated proteins VirB1 and VirB4. Based on the homology between VirB1 and VirB4 with the CagE N-, and C-terminus, respectively, we suggested earlier that CagE may represent a fusion product of a VirB1- and a VirB4-like amino acid sequence [19]. A scenario where the origin of the *cagE*^+^ serotype b *A. actinomycetemcomitans* strains is a recombination event on the chromosome, generating a fusion of parts of the genes encoding *virB1* and *virB4* (as illustrated in Figure 4), is plausible considering that chimeric proteins do exist in a number of bacterial T4SS gene clusters. For example, it was reported that *H. pylori* VirB3 and VirB4 is a fusion product, i.e., the first 150 amino acids of VirB4 have weak similarity with VirB3 although the motifs are conserved [31]. Similarly, a Western blot assay indicated a CagE-like protein pattern when prototypical *virB3* and *virB4* genes of *A. tumefaciens* were fused together and expressed [32]. Chimeric proteins are also encoded in a number of T4SS gene clusters of other species, including VirB3–VirB4 in *Campylobacter jejuni* [33,34], VirB1–VirB8 in *Bordetella pertussis* [35], and VirB11–VirD4 (MagB11–MagB12) in at least one strain of *A. actinomycetemcomitans* [25]. Moreover, observations with *H. pylori* are consistent with the notion that T4SS gene clusters can include regions that are prone to genetic rearrangements, resulting in the disruption or activation of the secretion system [36]. Results from our present work show that CagE and VirB1/VirB4 can be encoded in the same *A. actinomycetemcomitans* strain, albeit, as supported by PCR, with the T4SS genes most likely encoded on plasmids. The carriage of plasmids encoding T4SS genes, such as *virB1* and *virB4,* has been demonstrated in some *A. actinomycetemcomitans* strains [25,26]. In contrast, *cagE* appears not to be encoded on plasmids. According to the sequences available in the NCBI database, no hitherto sequenced *A. actinomycetemcomitans* plasmid carries a *cagE* gene locus. We were unable to detect by PCR the presence of a T4SS-encoding plasmid in the *cagE*-positive serotype b strain HK1651, which was earlier reported [25]. The reason for this discrepancy is not known but may reflect the possibility that this plasmid was lost in the strain preserved in our stocks upon repeated in vitro cultivation. The loss of plasmids of *A. actinomycetemcomitans* strains during in vitro cultivation is a phenomenon that has been reported earlier, albeit then related to growth in an antibiotic free medium [37]. 

Taken together, our present results further support the usefulness of the *cagE* gene as a potential diagnostic marker in the risk assessment of the development of attachment loss among young individuals. We conclude that *cagE* positive *A. actinomycetemcomitans* strains of serotype b among periodontitis patients living in Sweden consist of the JP2 and non-JP2 genotypes with phenotypic characteristics similar to the ones seen for the JP2 genotype strains but with a leukotoxin promoter region lacking the 530-bp deletion. Their origin, evolution, and extent of genetic similarity will be further explored by whole genome sequencing. 

## 4. Materials and Methods

### 4.1. Collection of A. actinomycetemcomitans Strains and Clinical Data Used in the Present Study 

For the present work, we used data from our microbiological analyses of 3459 subgingival plaque samples, collected from 1445 patients during 15 years (2000–2014) that included 337 ‘younger’ patients (≤35 years of age) and 1108 ‘older’ patients (>35 years of age) [27]. At the specialist clinics, it is recommended that microbial analysis is performed to study the microbial biofilm profiles of individuals ≤35 years affected by periodontal attachment loss, and of patients >35 years with rapidly progressive periodontitis, not responding to conventional periodontal therapy. The samples were sent from the Specialist Clinic of Periodontology at the Dental School in Umeå, Sweden, and from external specialist dental clinics throughout Sweden to be analyzed at the laboratory for microbiological diagnostics, Dental School, Umeå. The samples were collected from individuals between 9 and 92 years of age that were all diagnosed with periodontitis and referred to specialist clinics for periodontal treatment. However, due to the many clinics involved and the retrospective nature of the present study, clinical and other parameters were not systematically reported in the patient information attached to the referral to the laboratory for microbiological diagnostics. Therefore, the classification of the patients was dichotomized only and was based on the old definition of early onset periodontitis, which distinguished patients ≤35 years versus those >35 years of age [38]. An *A. actinomycetemcomitans* strain was collected and isolated from 347 patients [27]. PCR characterization revealed that 118 (34.0%) of the *A. actinomycetemcomitans* strains were serotype b, and 17 (14.4% of the serotype b strains) were characterized by 530-bp deletion in the promoter region of the leukotoxin gene operon (JP2 genotype). Among these 118 serotype b strains, we were able to cultivate and characterize 116 for use in the present study: 100 non-JP2 genotype and 16 JP2 genotype. For the present work, each of these 116 unique *A. actinomycetemcomitans* strains was combined with recorded clinical data, i.e., the age group of the patient (>35 or ≤35 years), and proportion of *A. actinomycetemcomitans* of the total cultivable microflora (TVC) in the sample.

### 4.2. Bacterial Strains and Growth Conditions

In the present work, we used a collection of 116 unique *A. actinomycetemcomitans* serotype b strains that were collected from periodontitis patients living in Sweden [27]. A list of these strains is presented in Table S1. The sampling of this collection and the subsequent characterization of the serotype, the AP-PCR genotype, and the leukotoxin promoter type (JP2/non-JP2 genotype) has been described earlier [27]. In the present study, 25 serotype b *A. actinomycetemcomitans* strains were used as reference, as they were subject to prior whole genome sequencing [15,19,39,40,41] (Table 1). Among these, five belong to a collection of oral *A. actinomycetemcomitans* strains previously reported on by Prof. Sirkka Asikainen: ANH9381, I23C, S23A, SCC1398 and SCC4092. Nine strains belong to the collection of serotype b *A. actinomycetemcomitans* strains, sampled from periodontitis patients living in Sweden: 133A1-08U, 196A1-10U, 115A-11U, 245-12U, 338A1-13U, 304A1-14U, 299A1-15U, 456A1-13U, and 520A-01U [28]. *A. actinomycetemcomitans* strains 443G, 486G, 575G, 605G, and 638G were sampled from a Ghanaian cohort of adolescents [6,42]. Finally, six type strains were included in the study: HK908 [29], HK909 [43], HK912 [29], HK921 [43], HK1651 [39], and Y4 [44,45]. All strains were cultured on blood agar plates (5% defibrinated horse blood, 5 mg of hemin/l, 10 mg of vitamin K/l, Columbia agar base) and incubated in air supplemented with 5% CO_2_ at 37 °C. 

### 4.3. DNA Isolation and Polymerase Chain Reaction Analysis

DNA templates for PCR and qPCR analysis were obtained by boiling a loopful of fresh *A. actinomycetemcomitans* colonies in 100 μl of water. *A. actinomycetemcomitans* genomic DNA to be used in AP-PCR was isolated using the GenElute™ Bacterial Genomic DNA kit (Sigma-Aldrich, St. Louis, MO, USA), following the manufacturer’s instructions. For the isolation of plasmids from *A. actinomycetemcomitans* strains, a QIAprep® Spin miniprep kit was used (QiaGen, Venlo, The Netherlands). Reaction mixtures for PCR were prepared using illustra™ PuReTaq™ Ready-To-Go™ PCR beads (GF Healthcare, Buckinghamshire, UK), whereas we used a KAPA SYBR® FAST qPCR Kit (KAPA Biosystems, Wilmington, MA, USA) for qPCR. The AP-PCR type was analyzed as earlier described [19,27], using the random sequence oligonucleotide OPB-3 (5′-AGTCAGCCAC-3′) (Invitrogen, Carlsbad, CA, USA) at 0.4 μmol/l and cycling conditions according to Dogan and coworkers [46]. The *cagE* gene was amplified by PCR as a 1020-bp DNA fragment, using a *cagE* forward primer (5’-GGATCCGTCCCTGAAATTTTATTAGCTTG-3’) and a *cagE* reverse primer (5-CTGCAGTTAAACGACCTTTAAACATTTTTTTA-3’) [20]. In qPCR analysis, *cagE* was detected as earlier described [19] using the *cagE*_F2 (5’-TGGATTGGGACAAGTGAACA-3’) and *cagE*_R2 (5’-CAATAATGGCTCGTGCAATATC-3’) primers to amplify a 623-bp internal fragment of the *cagE* gene. A ≈630-bp fragment of the lytic transglycosylase, *virB1* gene was amplified using a *cagE* forward primer and a *virB1* reverse primer (5’-GTTTTTAATCAATCTTCCTGATTG-3’). The amplification of the ATP:ase-encoding *virb4* gene, as a ≈900-bp DNA fragment, was carried out by PCR or qPCR using the *virB4* forward primer (5’-GTGCAGAAGCCTGTATTCGTGC-3’), and the *virB4* reverse primer (5’-CCAGTCATTAGTGGCTTCGCC-3’). The *magB01*-F (5’-GCCATCTACTACGCCTATCGC-3’) and *ssb*-R (5’-TTATCGCCGTCAAGCGGAAG-3’) primers [25] were used in PCR to assess the presence of plasmids encoding T4SS genes. PCR cycling conditions were 94 °C for 1 min, followed by 35 cycles of 94 °C for 30 sec, 54 °C for 30 sec, and 72 °C for 1 min, and then finally 72 °C for 7 min. The cycling conditions for qPCR were 95 °C for 10 min, followed by 45 cycles of 95 °C for 10 sec, 54 °C for 5 sec, and 72 °C for 22 sec. The complete genome sequences of serotype b strains SCC1398 (VirB1; GenBank accession KND83482), and I23C (VirB4; KOE53154) [40] were used as reference in oligonucleotide synthesis. 

### 4.4. Statistical Analysis and Image Processing

The rank test was used to calculate the strength of the association between the *A. actinomycetemcomitans* *cagE* and JP2 genotypes and proportion of TVC in subgingival plaque samples (IBM SPSS Statistics for Windows, Version 25.0, Armonk, New York). An odds ratio (OR) was used to quantify the strength of the association between the *A. actinomycetemcomitans cagE* genotype and age group (MedCalc for Windows, MedCalc Software, Ostend, Belgium). No normalization of the data or test unit was used in the present work.

### 4.5. Ethical Considerations

All procedures were conducted according to the guidelines of the local ethics committee at the Medical Faculty of Umeå University, which are in compliance with the Declaration of Helsinki (64^th^ WMA General Assembly, Fortaleza, October 2013). The characterization of the *A. actinomycetemcomitans* strains was made utilizing clinical samples from patients visiting the Specialist Clinic of Periodontology at the Dental School in Umeå. Data from specific strains were grouped in relation to age (>35 or ≤ 35 years) and could not be traced to a specific individual.

## Figures and Tables

**Table 1:** Genotyping of *A. actinomycetemcomitans* serotype b strains (n = 25) that were earlier subjected to whole genome sequencing.

Strain	Origin ^a^	*virB1* ^b^	*virB4* ^b^	JP2 ^c^	*cagE* ^d^	AP-PCR ^e^
ANH9381	Finland/Caucasian	+	+	-	-	2
HK908	Denmark/USA	+	+	-	-	2
HK909	Denmark	-	-	+	+	1
HK912	Denmark/USA	-	-	-	+	1
HK921	Denmark/USA	-	-	+	+	1
HK1651	Denmark/Ghana	-	-	+	+	1
I23C	Finland/Caucasian	+	+	-	-	2
S23A	Finland/Caucasian	+	+	-	-	2
SCC1398	Finland/Caucasian	+	+	-	-	other
SCC4092	Finland/Caucasian	+	+	-	-	other
Y4	USA	+	+	-	-	other
133A1-08U	Sweden^f^	-	-	+	+	1
196A1-10U	Sweden	-	-	+	+	1
115A-11U	Sweden	-	-	+	+	1
245-12U	Sweden	-	-	+	+	1
338A1-13U	Sweden	-	-	+	+	1
304A1-14U	Sweden	-	-	+	+	1
299A1-15U	Sweden	-	-	+	+	1
456A1-13U	Sweden	-	-	+	+	1
520A-01U	Sweden	-	-	+	+	1
443G	Sweden/Ghana	-	-	-	+	1
486G	Sweden/Ghana	-	-	-	+	1
575G	Sweden/Ghana	-	-	-	+	1
605G	Sweden/Ghana	-	-	-	+	1
638G	Sweden/Ghana	-	-	-	+	1

^a^ Geographic location of laboratories from where strains were obtained/origin of donor (where known)**;** ^b^ determined by PCR as described in Materials and Methods; ^c^ previously determined by PCR [14,28,29]; ^d^ previously determined by PCR [19]; ^e^ previously determined by AP-PCR [19], or deduced in the present work; ^f^ Sweden residents unless specified otherwise.

**Table 2:** Inverse relationship in the carriage of *cagE* and *virB4*. Presence of chromosomal *cagE* and *virB4* genes in serotype b strains of *A. actinomycetemcomitans* (n = 116) in different AP-PCR genotypes. The number of strains and percent (%) of all strains are indicated. The *cagE*-positive strains all (100%) belong to AP-PCR type 1 and lack the *virB4* gene.

	AP-PCR Type 1	AP-PCR Type 2	Other (Types 3–11)
***cagE^+^/virB4^-^***	49 (42.2)	0 (0)	0 (0)
***cagE^-^/virB4^+^***	0 (0)	11 (9.5)	15 (12.9)
***cagE^+^/virB4^+^***	0 (0)	0 (0)	0 (0)
***cagE^-^/virB4^-^***	0 (0)	14 (12.1)	27 (23.3)
**All strains**	49 (42.2)	25 (21.6)	42 (36.2)

**Table 3:** Age-associated distribution of the *cagE* genotype of serotype b. The prevalence of *cagE*-positive and *cagE*-negative strains among the *A. actinomycetemcomitans* serotype b strains (n = 116) sampled from young (≤35 yr; n = 62) and from old patients (>35 yr; n = 54). The numbers and percentages (%) of young, old, and all patients are indicated. The prevalence of *cagE*-positive strains was significantly higher (*p* < 0.001; odds ratio (OR) = 10.5, 95% CI: 4.2–26.1) in the young compared to old patients.

	Young Patients	Old Patients	All Patients
*cagE* ^+^	40 (64.5)	9 (16.7)	49 (42.2)
*cagE* ^-^	22 (35.5)	45 (83.3)	67 (57.8)

## Supplementary Materials

The following are available online at https://www.mdpi.com/2076-0817/8/3/153/s1. Table S1. The 116 *A. actinomycetemcomitans* serotype b strains, collected from periodontitis patients living in Sweden, and which were used in the present work.
