# Supplementary material for: Genetic Profiling of Aggregatibacter actinomycetemcomitans Serotype B Isolated from Periodontitis Patients Living in Sweden
Source: Pathogens. 2019 Sep 17;8(3):153. doi: 10.3390/pathogens8030153 (PMC6789814; doi:10.3390/pathogens8030153)
Supplement: Supplementary file 1 [file pathogens-08-00153-s001.pdf]

Supplementary Material

Table S1. The 116 *A. actinomycetemcomitans* serotype b strains, collected from periodontitis patients living in Sweden, and which were used in the present work. The *cagE*, JP2, and *virB4* genotypes, and AP-PCR type, is indicated for each strain.

**Strain isolate**		***cagE* genotype[1]**	**JP2 genotype[2]**	***virB4* genotype[3]**	**AP-PCR type[4]**
011 U[5]	908-00[6]	+	-	-	1
012 U	612-02	-	-	-	other
014 U	114-05	-	-	+	2
027 U	699-02	-	-	+	other
029 U	413-03	-	-	-	2
035 U	413-02	-	-	+	other
040 U	547-04	-	-	-	other
054 U	760-03	-	-	+	other
062 U	68-01	-	-	-	2
066 U	762-02	-	-	-	other
067 U	71-04	-	-	-	other
073 U	622-02	-	-	+	other
078 U	496-02	-	-	+	2
080 U	566-03	-	-	-	2
089 U	167-04	+	-	-	1
099 U	625-03	-	-	-	other
107 U	731-02	-	-	+	other
108 U	805-03	-	-	-	other

112 U	704-01	-	-	-	other
128 U	132-02	-	-	-	other
134 U	520A-01	+	+	-	1
135 U	780-03	+	-	-[7]	1
136 U	247-04	+	-	-	1
137 U	700-01	-	-	+	2
138 U	570-02	+	-	-	1
139 U	179-01	+	-	-	1
141 U	644-03	-	-	-	other
143 U	112-04	+	-	-	1
144 U	467-04	+	-	-	1
145 U	96-05	-	-	-	2
146 U	54-06	+	-	-	1
147 U	235-06	+	-	-[7]	1
148 U	70-08	-	-	-	other
149 U	133A1-08	+	+	-	1
152 U	879-00	+	-	-	1
153 U	746-02	-	-	+	other
160 U	700-02	-	-	-	other
161 U	295-03	-	-	-	2
167 U	287-09	+	-	-	1
172 U	380-09	+	-	-	1
173 U	399-09	-	-	+	other
177 U	582-09	-	-	-	2

179 U	595-09	-	-	-	other
184 U	17-10	+	-	-	1
185 U	38	-	-	+	2
186 U	090A-10	+	+	-	1
189 U	135-20	-	-	-	other
194 U	196A1-10	+	+	-	1
195 U	214-10	-	-	-	other
196 U	248-10	-	-	-	2
197 U	249-11	+	-	-	1
200 U	364-10	-	-	-	other
201 U	490-10	-	-	-	2
204 U	653-10	+	-	-	1
208 U	73-11	-	-	-	other
209 U	115A-11	+	+	-	1
212 U	173-11	+	-	-	1
214 U	187-11	+	-	-	1
219 U	349-11	-	-	-	other
221 U	325B-11	+	+	-	1
226 U	418-11	-	-	-	other
234 U	629-11	-	-	+	2
239 U	47-12	-	-	-	other
240 U	71-12	-	-	+	other
244 U	115-12	+	-	-	1
248 U	246A1-04	+	+	-	1

251 U	298-12	+	-	-	1
255 U	499-12	-	-	+	2
260 U	542-12	-	-	-	other
261 U	549-12	-	-	+	other
262 U	557A1-12	+	+	-	1
266 U	576-12	-	-	-	other
275 U	713-12	-	-	-	2
276 U	722-12	+	-	-	1
279 U	508-07	-	-	-	other
280 U	20-03	-	-	-	other
281 U	233-04	+	-	-	1
282 U	77-02	-	-	-	2
290 U	336-08	-	-	-	2
296 U	BLA1-08	+	+	-	1
302 U	051A1-13	-	-	+	other
307 U	074A1-13	-	-	+	other
310 U	107A1-13	-	-	-	2
311 U	135A1-13	-	-	+	2
314 U	181A1-13	-	-	+	2
317 U	338A1-13	+	+	-	1
318 U	342A1-13	+	+	-	1
320 U	371A1-13	-	-	+	2
321 U	373A1-13	+	-	-	1
327 U	408A1-13	+	+	-	1

329 U	412A1-06	-	-	-	2
335 U	456A1-13	+	+	-	1
346 U	654B1-06	-	-	-	other
348 U	012A1-14	+	-	-	1
352 U	065A1-14	+	-	-	1
354 U	121A1-14	+	-	-	1
356 U	136A1-14	-	-	+	other
358 U	138A1-14	+	-	-	1
360 U	168A1-14	+	-	-	1
364 U	215A2-14	+	-	-	1
374 U	346A1-14	+	-	-	1
376 U	381A1-14	-	-	-	2
384 U	478A1-14	-	-	+	other
391 U	588A1-14	-	-	-	other
393 U	613A1-14	+	-	-	1
398 U	671A1-14	-	-	-	other
400 U	688A1-14	-	-	+	other
403 U	717A1-14	-	-	-	other
405U	742A1-14	-	-	+	other
407 U	304A1-14	+	+	-	1
408 U	361A1-14	+	+	-	1
409 U	698A1-14	+	+	-	1
410 U	197A1-13	-	-	+	2
411 U	388A1-14	+	-	-	1

413 U	069A1-13	-	-	+	2
415 U	220A1-14	+	-	-[7]	1

[1] Previously determined [1], or deduced in the present work as described in Materials and methods

[2] Determined earlier [2]

[3] Presence of chromosomal *virB4* gene determined as described in Materials and methods

[4] Previously determined [2], or deduced in the present work as described in Materials and methods

[5]Identification number in our collection of *A. actinomycetemcomitans* isolates sampled from periodontitis patients living in Sweden

[6]Name of isolate

[7]T4SS determinants on plasmid according to PCR as described in Materials and methods

References

1. Johansson, A.; Claesson, R.; Höglund Åberg, C.; Haubek, D.; Oscarsson, J. The *cagE* gene sequence as a diagnostic marker to identify JP2 and non-JP2 highly leukotoxic *Aggregatibacter actinomycetemcomitans* serotype b strains. *J Periodontal Res* **2017**, *52*, 903-912.
2. Claesson, R.; Höglund-Åberg, C.; Haubek, D.; Johansson, A. Age-related prevalence and characteristics of *Aggregatibacter actinomycetemcomitans* in periodontitis patients living in Sweden. *J Oral Microbiol* **2017**, *9*, 1334504.
